# Supplementary material for: Evidence for white matter intrinsic connectivity networks at rest and during a task: A large-scale study and templates
Source: Netw Neurosci. 2025 Oct 30;9(4):1221–44. doi: 10.1162/NETN.a.29 (PMC12594490; doi:10.1162/NETN.a.29)
Supplement: Supplementary file 1 [file netn-9-4-1221-s001.pdf]

**Supplementary figures**

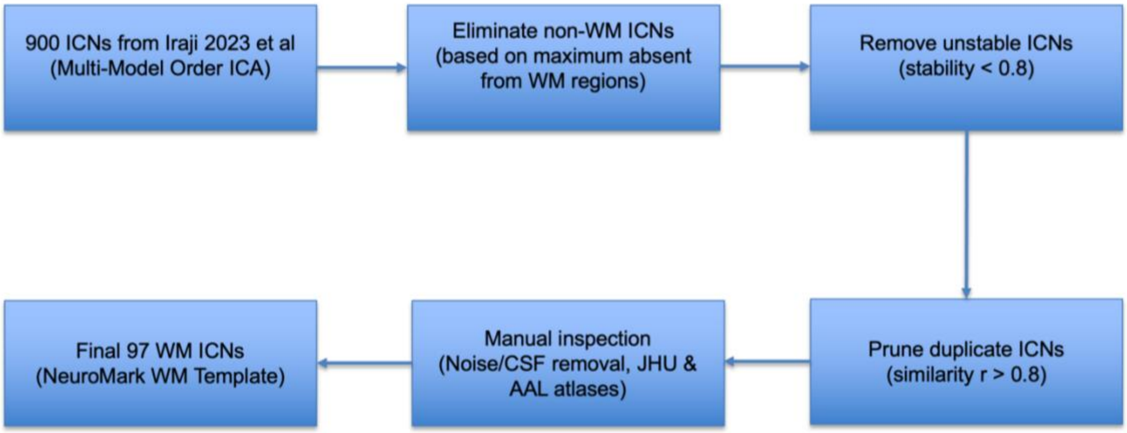


A)

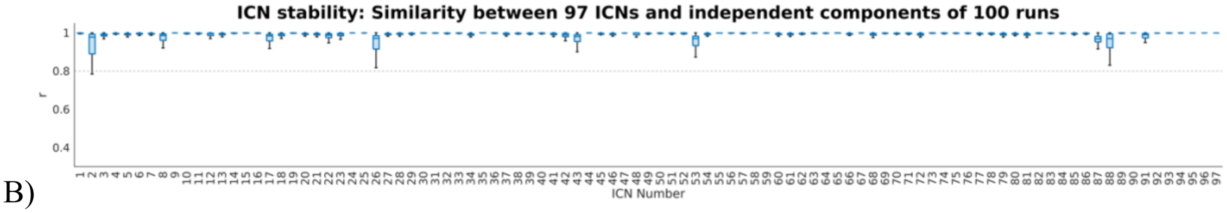


B)

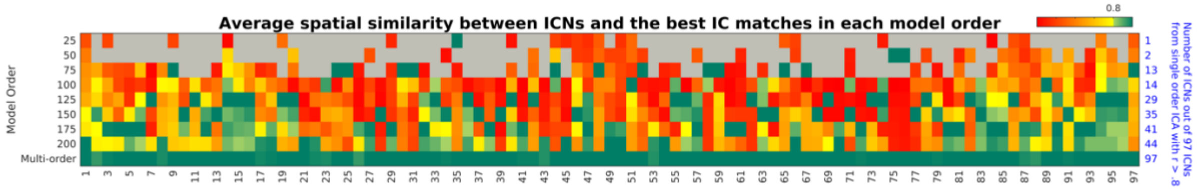


C)

Supplementary Figure 1: Workflow for WM ICN Template Creation and Evaluation of Model-Order Stability. A) Flowchart indicating the pipeline for generation of WM 97 ICN template. B) Multi-model order stability i.e., the spatial similarity of 97 WM ICNs with corresponding independent components (IC) across 100 group-level multi-model-order spatial ICA runs on different halves of the QC-passed dataset. C) In single-model-order and multi-model-order ICA, the average spatial similarity is calculated between each independent component network (ICN) and its best-matching ICs across 100 runs within each model order (including the combined multi-model-order case). The blue numbers shown on the right represent the count of ICNs identified at

each model order based on a spatial similarity threshold of 0.8, which corresponds to a stability coefficient of 0.8.

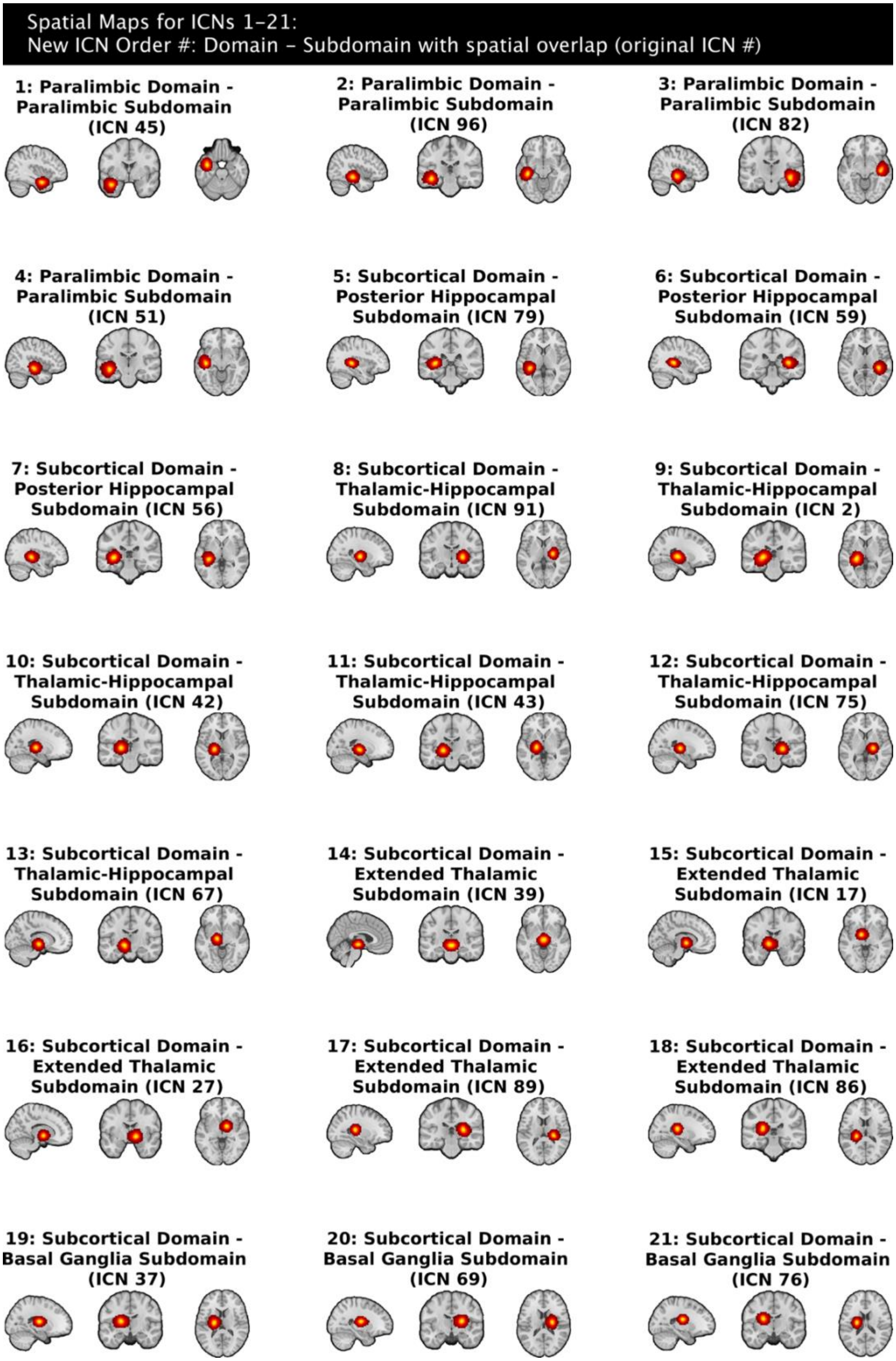
Spatial Maps for ICNs 1–21:
New ICN Order #: Domain – Subdomain with spatial overlap (original ICN #)
1: Paralimbic Domain - Paralimbic Subdomain (ICN 45)
2: Paralimbic Domain - Paralimbic Subdomain (ICN 96)
3: Paralimbic Domain - Paralimbic Subdomain (ICN 82)
4: Paralimbic Domain - Paralimbic Subdomain (ICN 51)
5: Subcortical Domain - Posterior Hippocampal Subdomain (ICN 79)
6: Subcortical Domain - Posterior Hippocampal Subdomain (ICN 59)
7: Subcortical Domain - Posterior Hippocampal Subdomain (ICN 56)
8: Subcortical Domain - Thalamic-Hippocampal Subdomain (ICN 91)
9: Subcortical Domain - Thalamic-Hippocampal Subdomain (ICN 2)
10: Subcortical Domain - Thalamic-Hippocampal Subdomain (ICN 42)
11: Subcortical Domain - Thalamic-Hippocampal Subdomain (ICN 43)
12: Subcortical Domain - Thalamic-Hippocampal Subdomain (ICN 75)
13: Subcortical Domain - Thalamic-Hippocampal Subdomain (ICN 67)
14: Subcortical Domain - Extended Thalamic Subdomain (ICN 39)
15: Subcortical Domain - Extended Thalamic Subdomain (ICN 17)
16: Subcortical Domain - Extended Thalamic Subdomain (ICN 27)
17: Subcortical Domain - Extended Thalamic Subdomain (ICN 89)
18: Subcortical Domain - Extended Thalamic Subdomain (ICN 86)
19: Subcortical Domain - Basal Ganglia Subdomain (ICN 37)
20: Subcortical Domain - Basal Ganglia Subdomain (ICN 69)
21: Subcortical Domain - Basal Ganglia Subdomain (ICN 76)

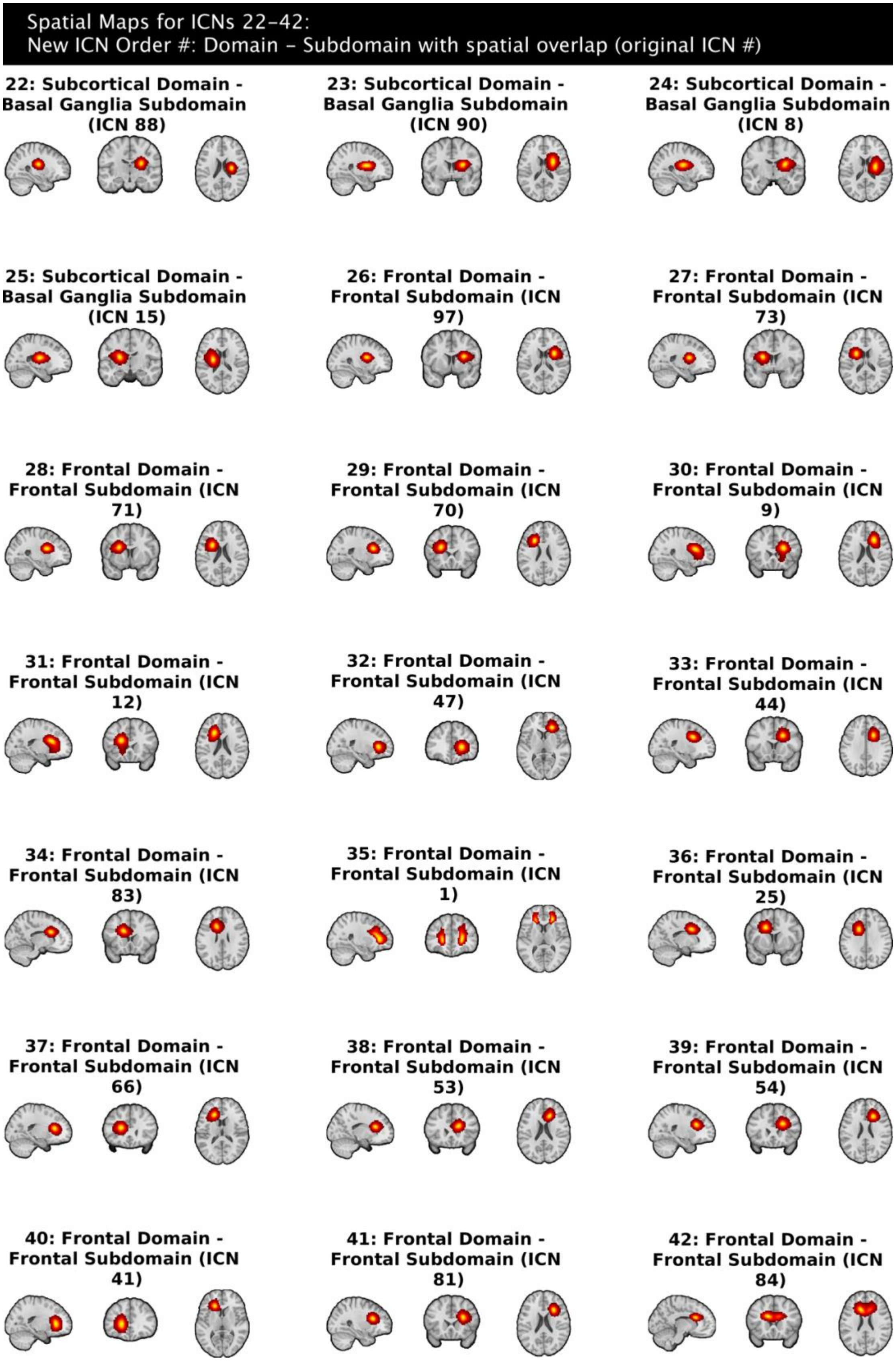
Spatial Maps for ICNs 22–42:
New ICN Order #: Domain – Subdomain with spatial overlap (original ICN #)
22: Subcortical Domain - Basal Ganglia Subdomain (ICN 88)
23: Subcortical Domain - Basal Ganglia Subdomain (ICN 90)
24: Subcortical Domain - Basal Ganglia Subdomain (ICN 8)
25: Subcortical Domain - Basal Ganglia Subdomain (ICN 15)
26: Frontal Domain - Frontal Subdomain (ICN 97)
27: Frontal Domain - Frontal Subdomain (ICN 73)
28: Frontal Domain - Frontal Subdomain (ICN 71)
29: Frontal Domain - Frontal Subdomain (ICN 70)
30: Frontal Domain - Frontal Subdomain (ICN 9)
31: Frontal Domain - Frontal Subdomain (ICN 12)
32: Frontal Domain - Frontal Subdomain (ICN 47)
33: Frontal Domain - Frontal Subdomain (ICN 44)
34: Frontal Domain - Frontal Subdomain (ICN 83)
35: Frontal Domain - Frontal Subdomain (ICN 1)
36: Frontal Domain - Frontal Subdomain (ICN 25)
37: Frontal Domain - Frontal Subdomain (ICN 66)
38: Frontal Domain - Frontal Subdomain (ICN 53)
39: Frontal Domain - Frontal Subdomain (ICN 54)
40: Frontal Domain - Frontal Subdomain (ICN 41)
41: Frontal Domain - Frontal Subdomain (ICN 81)
42: Frontal Domain - Frontal Subdomain (ICN 84)

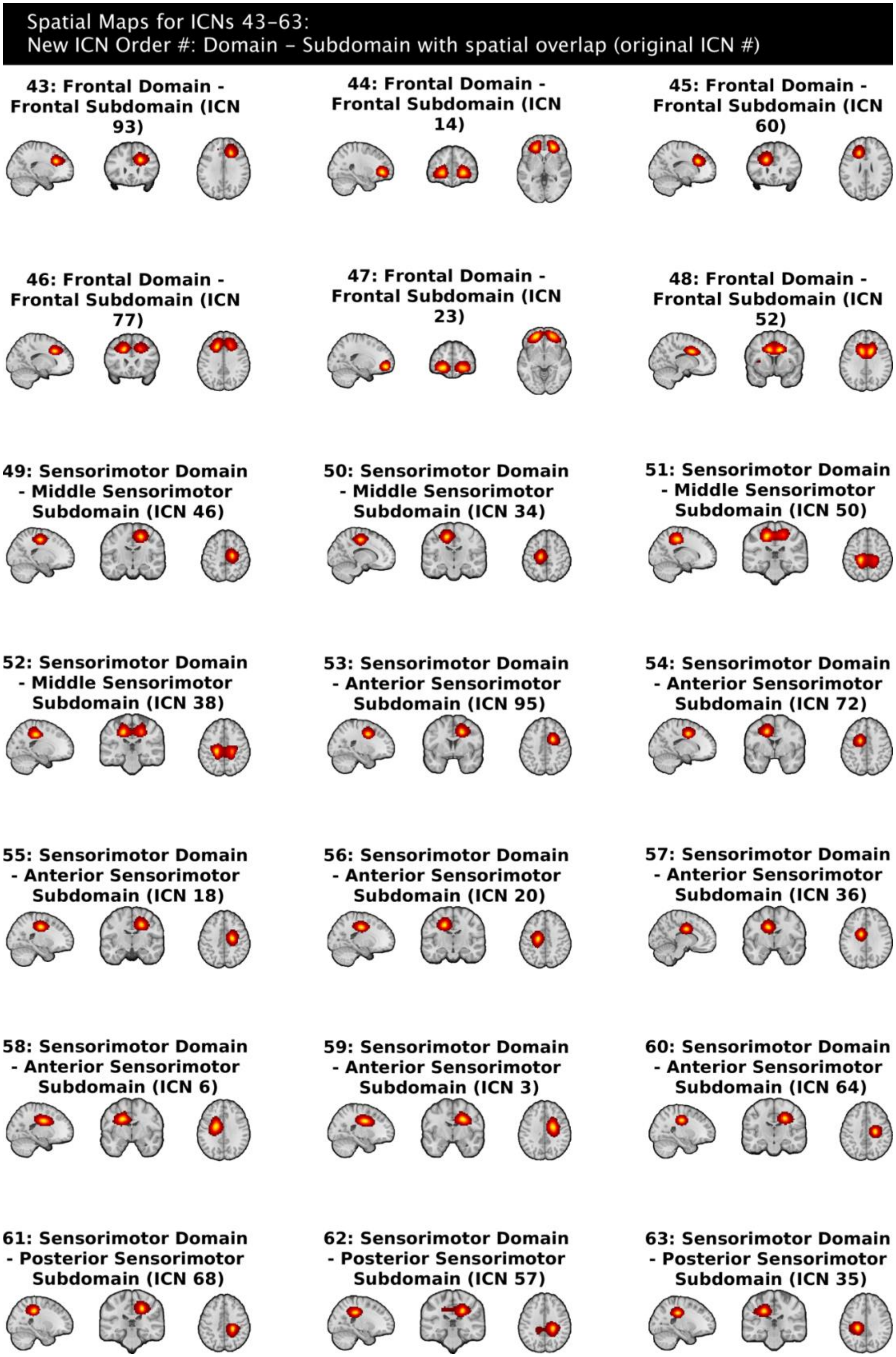
Spatial Maps for ICNs 43-63:
New ICN Order #: Domain – Subdomain with spatial overlap (original ICN #)
43: Frontal Domain - Frontal Subdomain (ICN 93)
44: Frontal Domain - Frontal Subdomain (ICN 14)
45: Frontal Domain - Frontal Subdomain (ICN 60)
46: Frontal Domain - Frontal Subdomain (ICN 77)
47: Frontal Domain - Frontal Subdomain (ICN 23)
48: Frontal Domain - Frontal Subdomain (ICN 52)
49: Sensorimotor Domain - Middle Sensorimotor Subdomain (ICN 46)
50: Sensorimotor Domain - Middle Sensorimotor Subdomain (ICN 34)
51: Sensorimotor Domain - Middle Sensorimotor Subdomain (ICN 50)
52: Sensorimotor Domain - Middle Sensorimotor Subdomain (ICN 38)
53: Sensorimotor Domain - Anterior Sensorimotor Subdomain (ICN 95)
54: Sensorimotor Domain - Anterior Sensorimotor Subdomain (ICN 72)
55: Sensorimotor Domain - Anterior Sensorimotor Subdomain (ICN 18)
56: Sensorimotor Domain - Anterior Sensorimotor Subdomain (ICN 20)
57: Sensorimotor Domain - Anterior Sensorimotor Subdomain (ICN 36)
58: Sensorimotor Domain - Anterior Sensorimotor Subdomain (ICN 6)
59: Sensorimotor Domain - Anterior Sensorimotor Subdomain (ICN 3)
60: Sensorimotor Domain - Anterior Sensorimotor Subdomain (ICN 64)
61: Sensorimotor Domain - Posterior Sensorimotor Subdomain (ICN 68)
62: Sensorimotor Domain - Posterior Sensorimotor Subdomain (ICN 57)
63: Sensorimotor Domain - Posterior Sensorimotor Subdomain (ICN 35)

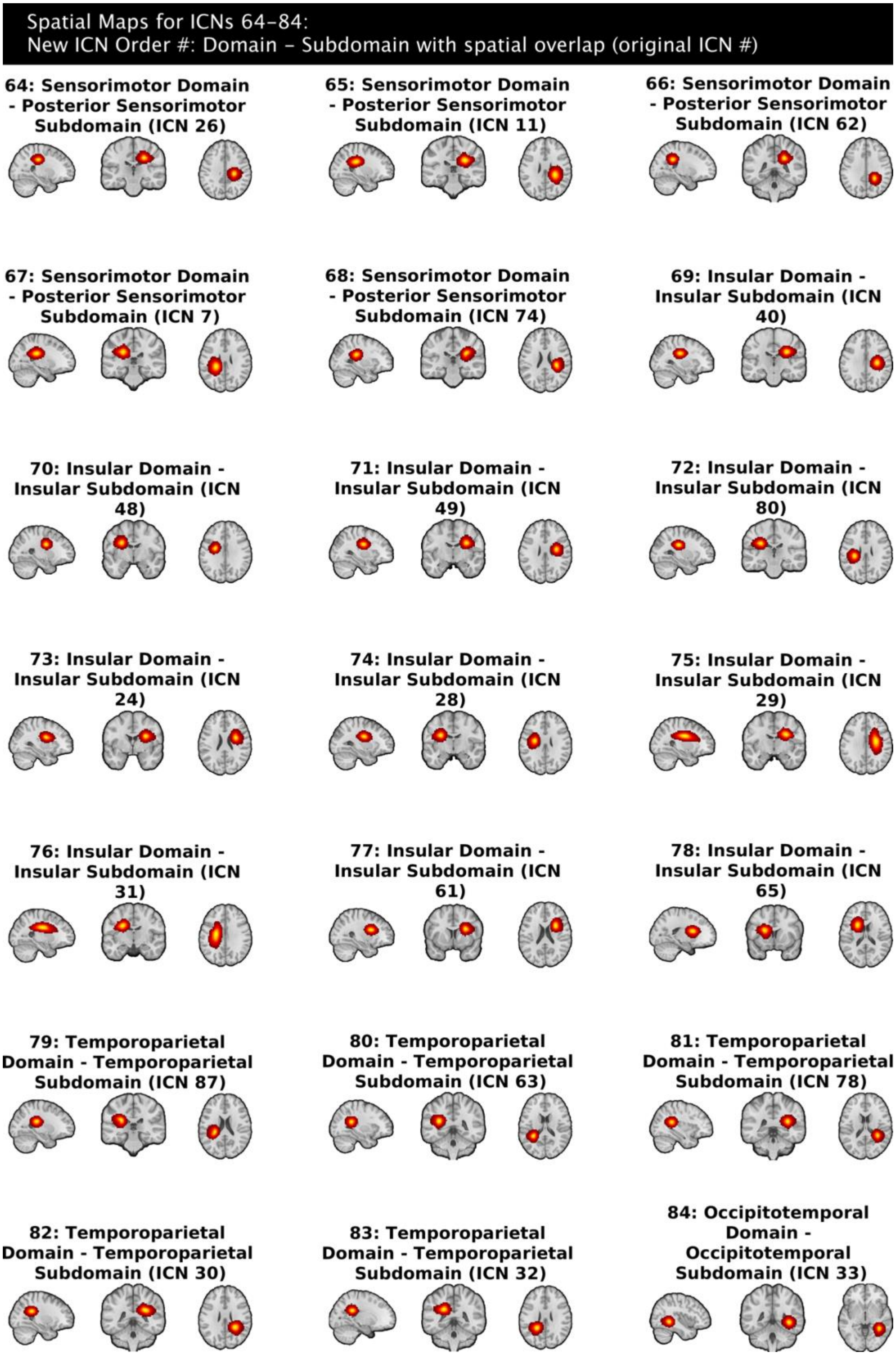
Spatial Maps for ICNs 64–84:
New ICN Order #: Domain – Subdomain with spatial overlap (original ICN #)
64: Sensorimotor Domain - Posterior Sensorimotor Subdomain (ICN 26)
65: Sensorimotor Domain - Posterior Sensorimotor Subdomain (ICN 11)
66: Sensorimotor Domain - Posterior Sensorimotor Subdomain (ICN 62)
67: Sensorimotor Domain - Posterior Sensorimotor Subdomain (ICN 7)
68: Sensorimotor Domain - Posterior Sensorimotor Subdomain (ICN 74)
69: Insular Domain - Insular Subdomain (ICN 40)
70: Insular Domain - Insular Subdomain (ICN 48)
71: Insular Domain - Insular Subdomain (ICN 49)
72: Insular Domain - Insular Subdomain (ICN 80)
73: Insular Domain - Insular Subdomain (ICN 24)
74: Insular Domain - Insular Subdomain (ICN 28)
75: Insular Domain - Insular Subdomain (ICN 29)
76: Insular Domain - Insular Subdomain (ICN 31)
77: Insular Domain - Insular Subdomain (ICN 61)
78: Insular Domain - Insular Subdomain (ICN 65)
79: Temporoparietal Domain - Temporoparietal Subdomain (ICN 87)
80: Temporoparietal Domain - Temporoparietal Subdomain (ICN 63)
81: Temporoparietal Domain - Temporoparietal Subdomain (ICN 78)
82: Temporoparietal Domain - Temporoparietal Subdomain (ICN 30)
83: Temporoparietal Domain - Temporoparietal Subdomain (ICN 32)
84: Occipitotemporal Domain - Occipitotemporal Subdomain (ICN 33)

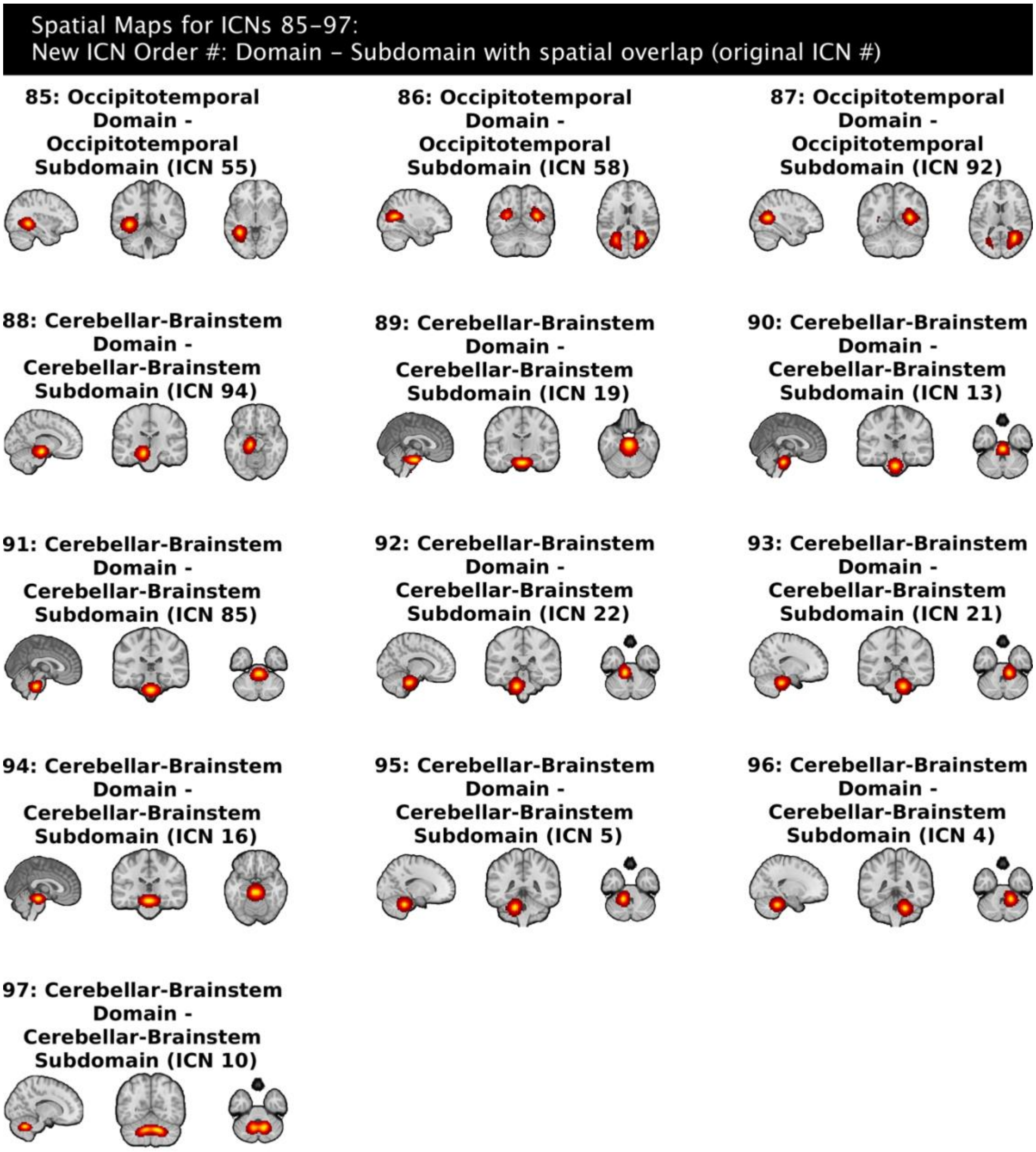


Supplementary Figure 2: Individual WM ICN spatial maps.

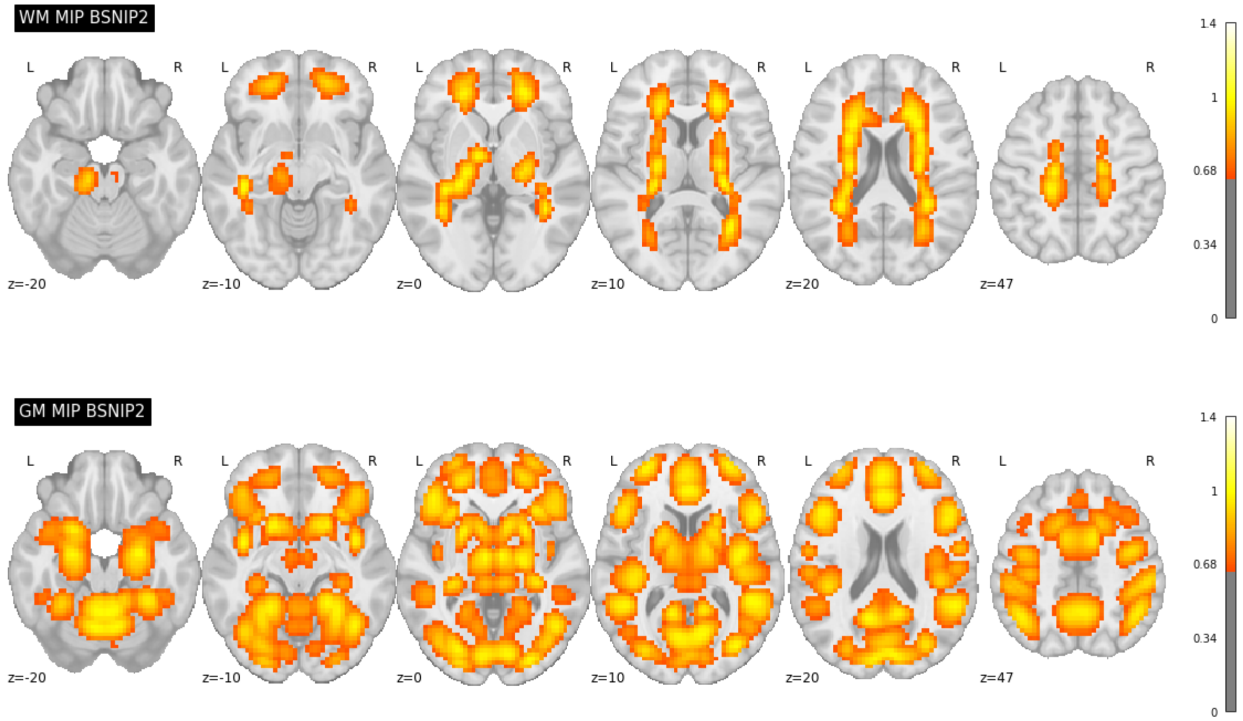


Supplementary Figure 3: MIPs of smoothed GM and WM ICNs derived from the BSNIP2 scICA output. The distinct functional connectivity patterns between GM and WM are evident, further reinforcing the inherent differences between these tissue types.
